# Correction: Abou-Elnour et al. Investigation of the Optical Properties for Quaternary Se_60−x_Ge_35_Ga_5_Sb*_x_* (*x* = 0, 5, and 10) Chalcogenide Glass. *Materials* 2022, *15*, 6403

**DOI:** 10.3390/ma16051875

**Published:** 2023-02-24

**Authors:** Huda Allah Abou-Elnour, M. B. S. Osman, M. Fadel, A. M. Shakra

**Affiliations:** 1 Physics Department, Faculty of Women for Arts, Science and Education, Ain Shams University, Cairo 11566, Egypt; 2 Environmental Research Department, National Institute of Occupational Health and Safety (NIOSH), Cairo 2208, Egypt; 3 Semiconductor Lab., Physics Department, Faculty of Education, Ain Shams University, Cairo 11341, Egypt

The authors would like to make a correction in a recently published paper [1].

**Error in Figure**

In the original publication, there was a mistake in:

(1) Figure 3a,b The transmittance and reflectance spectra of the evaporated film at a thickness of 450 nm as published. Figure 3a was replaced by mistake with a figure with a different scale in the last version of editing, while in Figure 3b, text that refers to each line was removed from the drawing graph to be similar to Figure 3a The corrected Figure 3a,b appears below.

(2) Figure 12 Plots of (*n*^2^ − 1)^−1^ versus (*hυ*)^2^ for Se_60−*x*_Ge_35_Ga_5_Sb*_x_* thin films. as published.

Figure 12 was replaced with another graph by mistake only in the last version of editing. The corrected Figure 12 appears below:

**Figure 12:**
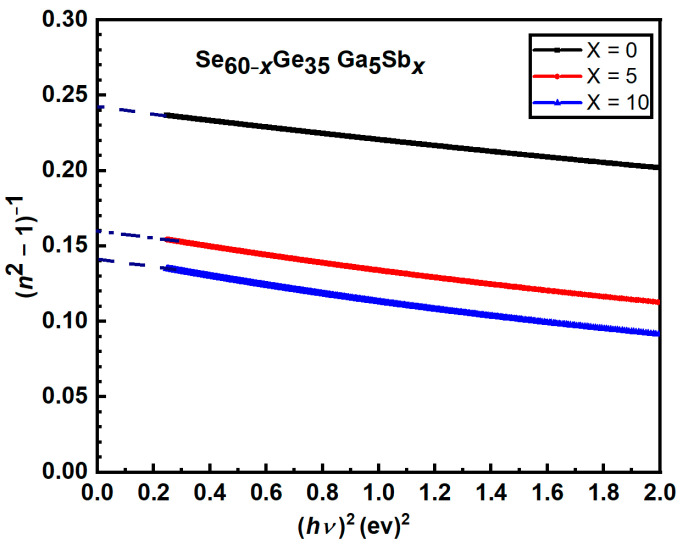
Plots of (*n*^2^ − 1) ^−1^ versus (*hυ*)^2^ for Se_60−*x*_Ge_35_Ga_5_Sb*_x_* thin films.

The authors apologize for any inconvenience caused and state that the scientific conclusions are unaffected.

## Figures and Tables

**Figure 3:**
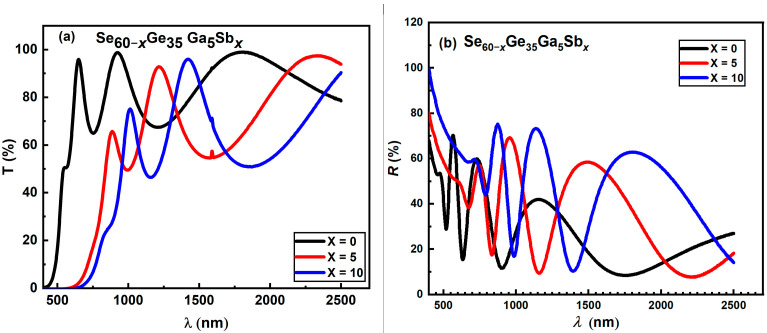
(**a**,**b**) The transmittance and reflectance spectra of the evaporated film at a thickness of 450 nm.
