# Supplementary material for: Investigation of the Optical Properties for Quaternary Se60−xGe35Ga5Sbx (x = 0, 5, and 10) Chalcogenide Glass
Source: Materials (Basel). 2022 Sep 15;15(18):6403. doi: 10.3390/ma15186403 (PMC9501473; doi:10.3390/ma15186403)
Supplement: Supplementary file 1 [file materials-15-06403-s001.zip › materials-1856792-supplementary.pdf]

Supportive information

Investigation of the Optical Properties for Quaternary $Se_{60-x}Ge_{35}Ga_5Sb_x$ (x= 0,5, and 10) Chalcogenide Glass

Huda Allah Abou-Elnour [1,2,*], M. B. S. Osman [1], M. Fadel [3] and A. M. Shakra [3]

1 Physics Department, Faculty of Women for Arts, Science and Education Ain Shams University, Cairo, Egypt
2 Environmental Research Department, National Institute of Occupational Health and Safety (NIOSH- Egypt)
3 Semiconductor Lab., Physics Department, Faculty of Education, Ain Shams University, Cairo, Egypt

* Correspondence: hudaallah.hse@gmail.com, hudaallah.said@women.asu.edu.eg ; Tel.: +20-10-0462-1948.

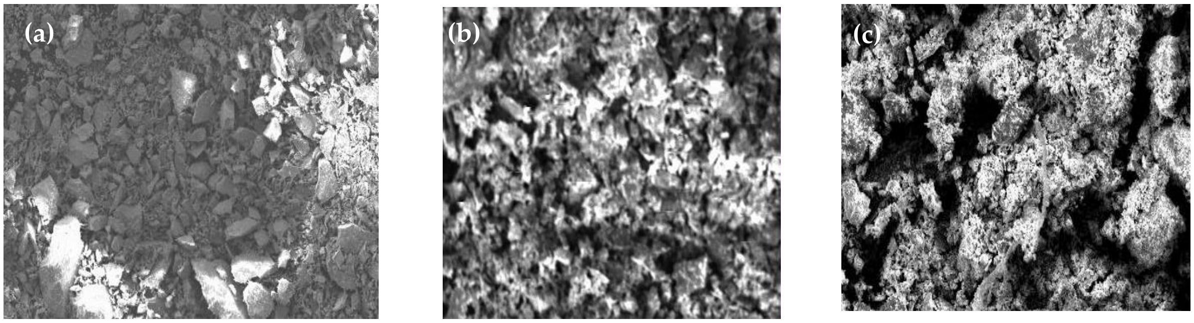

Figure S 1. SEM images of (a) $Se_{60}Ge_{35}Ga_5$ *, (b)* $Se_{55}Ge_{35}Ga_5Sb_5$*, (c)* $Se_{50}Ge_{35}Ga_5Sb_{10}$.
